# Fecal Calprotectin for predicting Relapse and Activity in Patients with Crohn’s Disease: A Meta-analysis

**DOI:** 10.5005/jp-journals-10018-1182

**Published:** 2016-12-01

**Authors:** Ying Zhuge, Qiu-Ping Huang, Qing Li, Jun-Shan Wang

**Affiliations:** 1 Department of Cardiology, Affiliated Shanghai First People’s Hospital, Shanghai Jiao Tong University, Shanghai, China; 2 Department of Internal Medicine, Affiliated Shanghai First People’s Hospital, Shanghai Jiao Tong University, Shanghai, China; 3 Department of Gastroenterology, Shanghai Tenth’s Hospital of Tongji University, Shanghai, China

**Keywords:** Crohn’s disease, Fecal calprotectin, Meta-analysis, Prognostic factor.

## Abstract

**Aims:**

We aimed to perform a meta-analysis of the predictive capacity of fecal calprotectin (FC) in activity and relapse of Crohn’s disease (CD).

**Materials and methods:**

MEDLINE, EMBASE, and the Cochrane Library databases were searched systematically. Pooled sensitivity, specificity, and other diagnostic indices were evaluated.

**Results:**

A total of 1,252 CD patients from 18 different studies were analyzed. The pooled sensitivity and specificity of FC at a cutoff value of 50 μg/g to predict activity of CD were 0.91 [95% confidence interval (CI): 0.87–0.95] and 0.47 (95% CI: 0.35–0.59) respectively. The pooled sensitivity and specificity of FC at a cutoff value of larger than 150 μg/g to monitor relapse of CD was 0.75 (95% CI: 0.67–0.82) and 0.71 (95% CI: 0.66–0.76) respectively. The area under the summary receiver operating characteristic (SROC) curve of FC for detecting CD activity was 0.78 (50 μg/g), 0.88 (100 μg/g), 0.85 (>150 μg/g), and the diagnostic odds ratio (DOR) was 10.21 (50 μg/g), 10.20 (100 μg/g), 11.68 (>150 μg/g) respectively.

**Conclusion:**

As a simple and noninvasive marker, FC is useful to predict the activity and relapse in CD patients, and the capacity of FC to predict CD activity was superior to its application in monitoring relapse of CD.

**How to cite this article:**

Zhuge Y, Huang Q-P, Li Q, Wang J-S. Fecal Calprotectin for predicting Relapse and Activity in Patients with Crohn’s Disease: A Meta-analysis. Euroasian J Hepato-Gastroenterol 2016;6(2):116-124.

## INTRODUCTION

Crohn’s disease (CD) is one of the major forms of idiopathic inflammatory bowel disease (IBD).^1^ Crohn’s disease is a chronic inflammatory disorder of the gastrointestinal tract, i.e., characterized by unknown etiology, accompanied with recurrent exacerbations and remissions. Crohn’s disease has a similar prevalence in both men and women, and the number of patients suffering from CD has been increasing globally in the last decades. The highest incidence of CD has been reported in North America and North Europe.^2^ It has been recognized that CD is a disabling disorder influencing health-related quality of life.

Medical intervention of CD includes conventional treatment, such as corticosteroids, immunomodulators, and biological agents. As tumor necrosis factor (TNF) is an important mediator of the development of CD in the digestive tract, biological agents, such as infliximab and adalimumab, specifically targeting TNF, have remarkably improved the outcome of CD patients in recent years.^3^ However, despite these improvements in the treatment of CD, there is still subclinical inflammation in the gut, leading to higher risk of relapse or recurrence.^2-4^ Thus, monitoring the activity of inflammation and identifying patients who may benefit most from conventional or biological therapy are of great interest. For years, the assessment of activity of CD is mainly based on a combination of symptoms, clinical findings, and endoscopy.^5^ These information are not always in accordance with the actual condition of CD due to the insufficient correlation between items of examination and disease pathology.

Ileocolonoscopy is the gold standard test for evaluating activity of intestinal inflammation, but its clinical use is severely limited by many disadvantages including patient intolerance, invasiveness, high cost, and time consumption. In order to overcome these issues, a number of laboratory noninvasive parameters have been assessed regarding their roles in monitoring activity of CD. The acute-phase reaction and releases of various proteins by immune cells, such as leukocytes are often associated with active inflammation in patients with CD, which enables the determination of CD activity by detecting the released proinflammatory factors in serum or other sources. Erythrocyte sedimentation rate (ESR), C-reactive protein (CRP),^6^ serum concentration of anti-TNF agents,^6-9^ and fecal calprotectin (FC)^10-15^ are currently being studied in patients with CD, and some of them exhibit good results in predicting treatment efficacy or activity of CD. Levesque et al^8^ have prospectively evaluated the relationship between serum infliximab concentration and CD activity, found that infliximab level below 2.8 to 4.6 μg/mL can be best predicted by a 70-point increase in Crohn’s disease activity index (CDAI), and concluded that infliximab concentration below 3 μg/mL may increase the likelihood of symptoms and inflammatory activity of CD. Previous study of Schoepfer et al^5^ demonstrated the relationship between the Simple Endoscopic Score for Crohn’s disease (SES-CD) and FC, CRP, and CDAI and reported that the overall accuracy for the detection of endoscopically active disease was 87% for calprotectin, 66% for increased CRP, and 40% for CDAI ≥ 150, indicating that FC correlates closest with SES-CD and therefore activity of CD. These studies proved that FC and serum concentration of anti-TNF agents may be superior to CRP and CDAI in predicting CD activity. In this study, we performed a meta-analysis and pooled data from available studies to evaluate the diagnostic value of FC in assessing the activity and relapse of CD.

## MATERIALS AND METHODS

A systematic review and meta-analysis of observational trials aiming to identify the predictive ability of FC for relapse and/or activity of CD were performed. The methodology presented below included inclusion and exclusion criteria, data sources, search strategies, study selection, and data extraction, outcome measures, assessment of quality of studies, and statistical analysis.

## Inclusion and Exclusion Criteria

Clinical studies that described the predictive roles of FC in monitoring activity or relapse of CD were eligible for inclusion. Other criteria for inclusion included studies with adult populations, articles written in English, and evaluating relationship between FC and CD. Studies not written in English, animal studies, studies without proper control setting, absence of abstract, insufficient data, or those focusing on quality of life were excluded.

## Data Sources

With the purpose of finding primary clinical studies, we systematically searched the following electronic databases for literature published from 1966 to August 2014: Medline (PubMed), the Cochrane central register of controlled trials, EMBASE, PubMed, EMBASE, the Cochrane Database of Systematic Reviews, and DARE. In addition, we also searched the websites of the British Society of Gastroenterology and European Crohn’s and Colitis Organization to collect sufficient information of the clinical studies. The network search engine Google scholar was also used to identify additional studies. If necessary, hand search was performed to find relevant articles.

## Search Strategies

The main strategy to select eligible studies was a broad systematic search using the PubMed database with the following keywords and Medical Subject Heading (MeSH) terms: (“calprotectin” [All Fields] OR “calprotectin” [All Fields] OR “leukocyte L1 antigen complex” [All Fields] OR “calgranulin” [All Fields]) AND (“Crohn disease” [MeSH Terms] OR “Crohn” [All Fields] OR “CD” [All Fields]). Literature identifications were also done in EMBASE database and the Cochrane database using the same search keywords or MeSH terms.

## Study Selection and Data Extraction

The process of search and selection of eligible studies was performed by two reviewers independently. The methods detailed in the Cochrane handbook were introduced during the screen and selection period to ensure the quality of selection.^16^ Titles of all selected studies were screened, and articles that did not meet the inclusion and exclusion criteria were excluded. The abstracts of the remaining articles were read to further identify inappropriate studies. The two reviewers viewed the full text of the papers ready for inclusion after previous selection steps and again using the eligibility criteria mentioned above. The baseline characteristic information of each included paper was recorded: Total number of patients, FC assay, cutoff value of FC, and standard of relapse. The data extraction was conducted by two reviewers independently applying a standard data extraction form, and then the information was cross-checked with each other. If there was an uncertainty of specific issue, a third reviewer was invited to solve the concern. For the included articles, data were also extracted on clinical outcomes.

## Quality Assessment

Two reviewers were employed for the assessment of methodological quality of each included study. The risks of bias detailed in the Cochrane handbook for diagnostic test accuracy review were assessed as following: Risks of bias of patient selection, index test, reference standard, and flow and timing.

## Statistical Analysis

The methods recommended in the Cochrane handbook for systematic reviews of diagnostic test accuracy were used in this meta-analysis.^17^ Reference-positive patients/total subjects were used to calculate the pretest probability of CD. The sensitivity and specificity of FC in a certain study were extracted or calculated using appropriate contingency tables. If there were potential problems in odds calculations for studies with sensitivities or specificities of 100%, then a value of 0.5 was added to all cells of trials that contained zero.^18^ Positive likelihood and negative likelihood were determined as functions of these summary estimates; the derived estimates of sensitivity, specificity, and respective variances were also used to construct a summary receiver operating characteristic (SROC) curve.^18^ The area under the ROC curve was used as an alternative global measure of test performance.^18^ Diagnostic odds ratio (DOR) and the area under the SROC curve (AUC) were calculated to evaluate the diagnostic performance of FC in patients with CD.

Diagnostic odds ratio and the AUC were computed to assess the prognostic performance of FC in CD patients. The formula for calculating DOR was presented as following: (sensitivity/[1 sensitivity])/([1 specificity]/specificity). A DOR of 1 indicates that the test fails to differentiate patients with active and inactive, responder and in-responder CD patients. A higher value stands for better test performance. An AUC of 1 equals a perfect test and 0.5 a completely uninformative test.^17^ A model of random effects at each threshold was used to establish the pooled sensitivity, and specificity, with relevant 95% confidence interval (CI). We also performed subgroup analysis to assess potential risk of bias regarding country, method, aim, and FC concentration.

The chi-square test or *Q*-statistic and Higgins *I*^2^ statistic were applied to detect possible heterogeneity. We considered that there was a statistically significant heterogeneity if p < 0.1. The percentage of *I*^2^ indicated the degree of heterogeneity, and 25, 50, and 75% represented a low, moderate, and high degree of heterogeneity respectively.^19^ Software Meta Disk v1.4 was used to carry out all the statistical computations. It was considered to be statistically significant if the p values were less than 0.05.

## RESULTS

After the initial search, a total of 278 articles were selected; 57 of them were eligible for the initial analysis after further review. However, during the process of full-text review and data extraction, another 39 studies were excluded: 12 studies were not relevant studies; 11 studies were excluded as they were reporting clinical treatment outcome instead of predicting CD activity or relapse; 6 studies were excluded as concentrations of FC used for prediction were insufficient; 3 studies were excluded as the patients overlapped with another study; 5 studies were excluded as they were review papers; and 2 studies were discarded as it was performed in pediatric patients. Therefore, 18 studies^5,10,12,14,20-33^ were included in the final analysis. The details of study selection is presented Flow Chart 1.

**Flow Chart 1:**
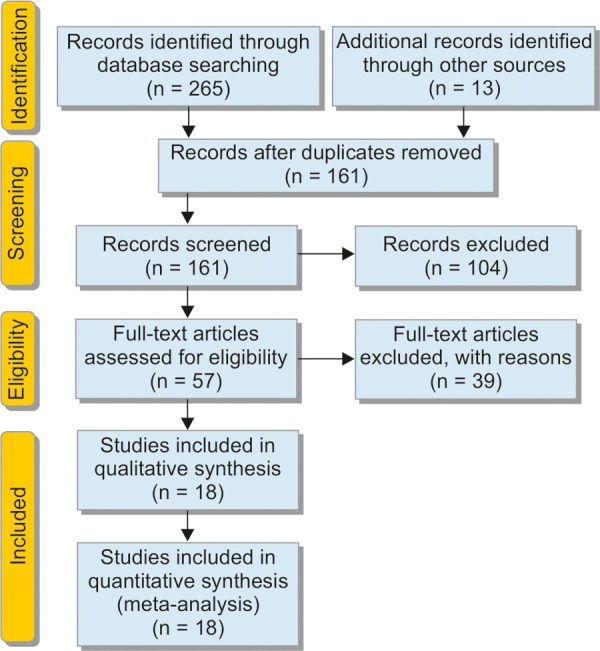
Study flow diagram

### Study Characteristics and Quality of Included Studies

**Fig. 1:**
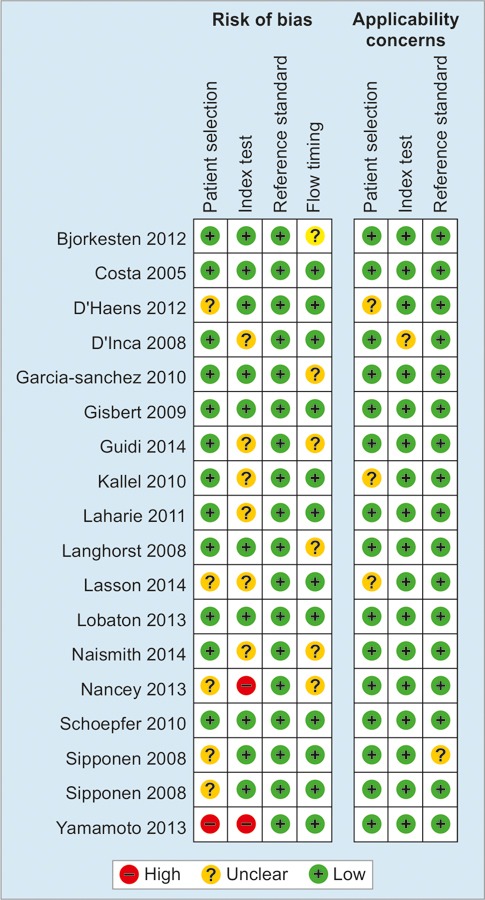
Summary of the methodological assessment of the included studies based on the Cochrane handbook

**Fig. 2:**
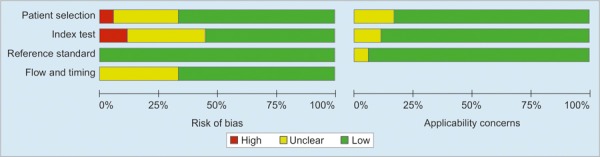
Risk of bias and applicability concerns graph: review authors’ judgments about each domain presented as percentages across included studies

The baseline clinical characteristics and main outcomes of these studies are listed in Table 1. In brief, these included studies were of prospective design, and the populations involved were all diagnosed as CD. However, the enrolled patients in different studies were not in the same condition. The study of Yamamoto et al^20^ enrolled participants with quiescent CD who had operation before, and the size of this study was relative small. Bjorkesten et al^23^ recruited anti-TNF-positive CD patients. Endoscopies and FC tests were conducted at the beginning of these studies and at certain time point. And endoscopies were used as the reference standards in these trials, although the reporting terms of results of endoscopies were not inconformity. Each item of risk of bias for individual studies is assessed and presented in Figures 1 and 2.

**Table 1:** **Table 1:** Characteristics of the included studies

														*Test results*							
*Study*		*Age*		*n*		*Site*		*FC assay*		*Reference standard*		*Cutoff (μg/g)*		*TP*		*FP*		*FN*		*TN*	
Langhorst et al^21^		15–70		43		Germany		ELISA		Endoscopy scoring method		30		33		7		0		3	
												240		27		2		6		8	
Sipponen et al^10,22^		19–70		106		Finland		PhiCal		CDEIS ≥ 3		50		64		20		6		16	
												100		57		11		13		25	
												200		49		3		21		33	
Schoepfer et al^5^		18–85		140		Switzerland		PhiCal		SES-CD ≥ 3		50		101		11		13		15	
												70		101		7		13		19	
Bjorkesten et al^23^		18–69		126		Finland		PhiCal		SES-CD ≥ 3		100		83		6		20		17	
D’Haens et al^24^		30–64		87		The Netherlands		PhiCal		SES-CD ≥ 2		250		29		8		19		31	
Nancey et al^25^		18–79		78		France		ELISA		SES-CD ≥ 3		100		33		25		5		15	
												250		27		9		11		31	
Yamamoto et al^20^		32 ± 2		20		Japan		ELISA		Rutgeerts ≥ 2		140		7		3		3		7	
Lobaton et al^26^		32–58		89		Spain		ELISA		CDEIS ≥ 3		274		36		11		1		41	
Kallel et al^27^		15–66		53		Tunisia		PhiCal		CDAI > 150		340		8		4		2		39	
Garcia-Sanchez et al^28^		27–54		66		Spain		PhiCal		CDAI ≥ 150		200		14		17		4		31	
Gisbert et al^29^		30–56		89		Spain		PhiCal		CDAI > 150		169		9		18		4		58	
D’Inca et al^30^		15–80		65		Italy		PhiCal		CDAI > 150		130		13		17		7		28	
Costa et al^31^		24–54		38		Italy		PhiCal		CDAI > 150		150		13		13		2		10	
Laharie et al^32^		15–69		65		France		ELISA		CDAI > 150		50		16		15		7		8	
												100		15		12		8		11	
Lasson et al^14^		17–63		30		Sweden		ELISA		Rutgeerts ≥ 2		100		11		11		2		6	
												200		8		9		7		6	
Naismith et al^12^		47 ± 16		92		UK		ELISA		CDAI > 150		240		8		21		2		61	
Guidi et al^33^		22–47		50		Italy		ELISA		Decrease of CDAI > 100		121		7		12		3		28	
												168		24		3		6		17	
Sipponen et al^10,22^		19–44		15		Finland		PhiCal		CDEIS ≥ 3		200		10		0		1		4	

ELISA: Enzyme-linked immunosorbent assay; N: Number of patients; CDEIS: Crohn’s disease endoscopic index of severity; TP: True positive; FP: False positive; TN: True negative; FN: False negative

### Diagnostic Accuracy

**Graph 1:**
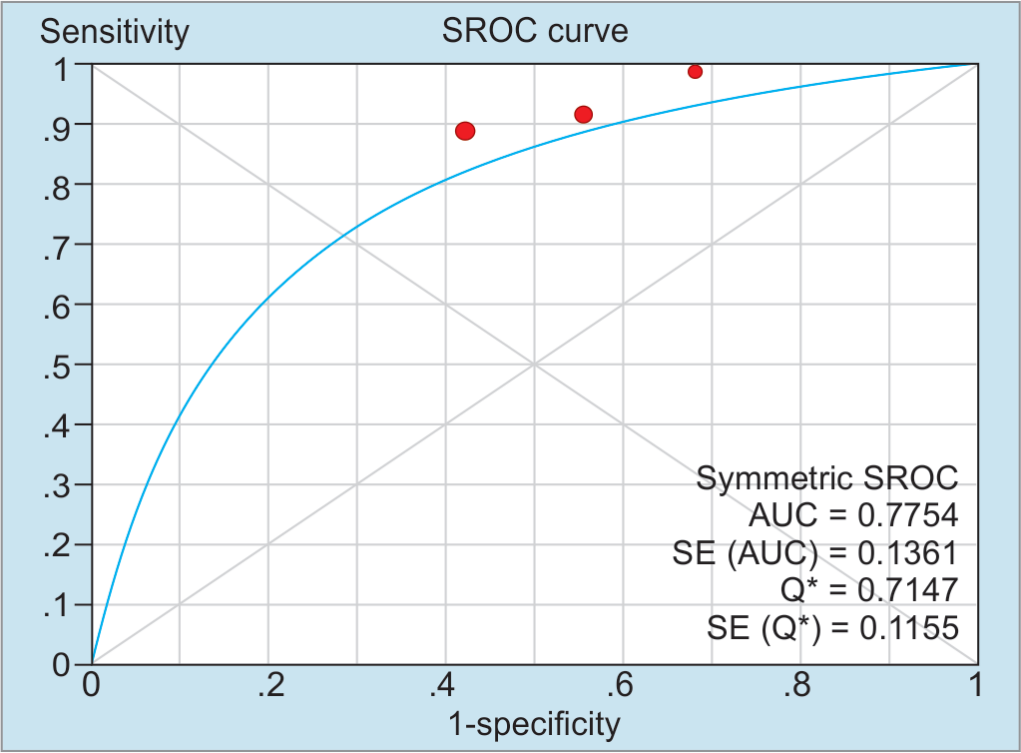
Summary receiver operating characteristic curve of FC assay in predicting CD activity at a cutoff value of 50 μg/g. Red spots denote the included studies of this pooled analysis

**Graph 2:**
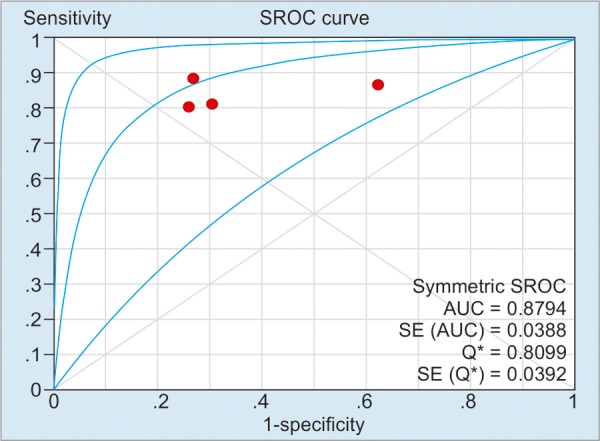
Summary receiver operating characteristic curve of FC assay in predicting CD activity at a cutoff value of 100 μg/g. Red spots denote the included studies of the pooled analysis

**Graph 3:**
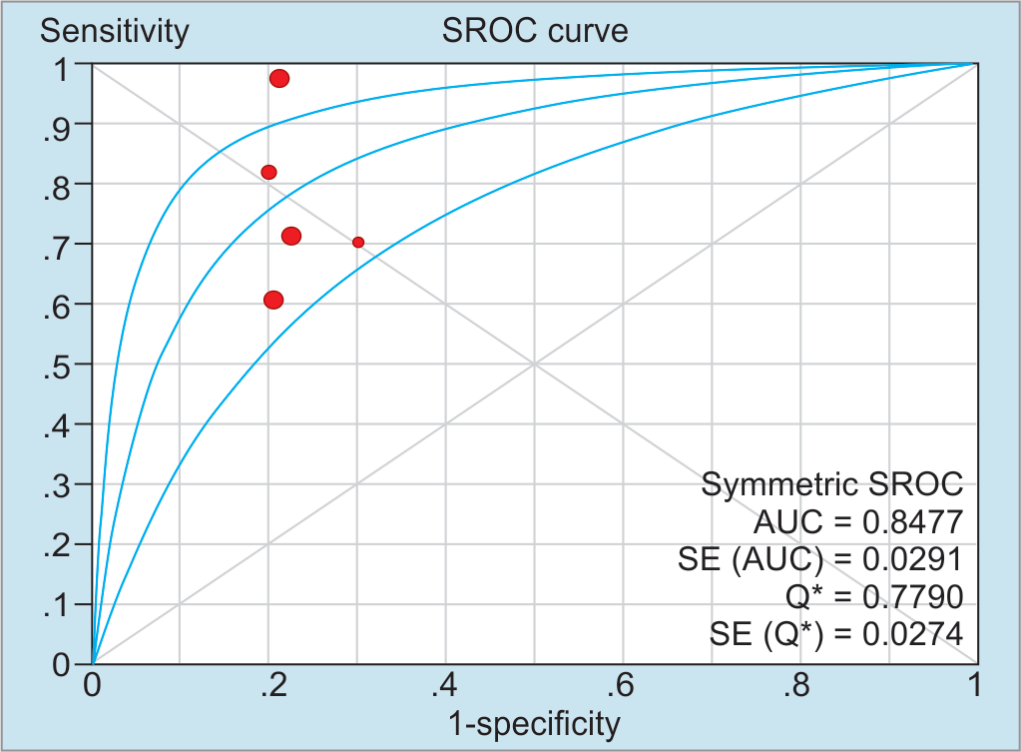
Summary receiver operating characteristic curve of FC assay in predicting CD activity at a cutoff value of >150 μg/g. Red spots denote the included studies of the pooled analysis

**Graph 4:**
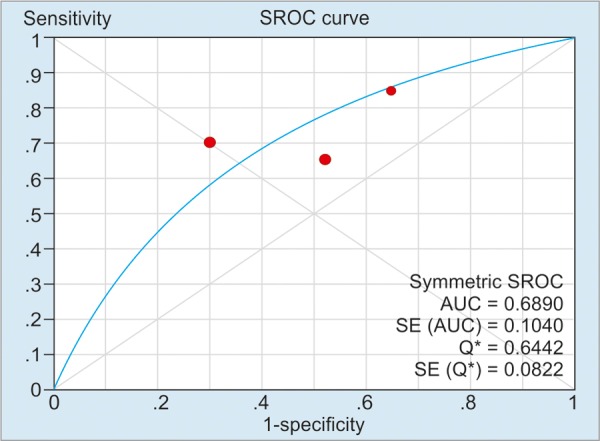
Summary receiver operating characteristic curve of FC assay in monitoring CD relapse at a cutoff value around 100 μg/g. Red spots denote the included studies of this pooled analysis

**Graph 5:**
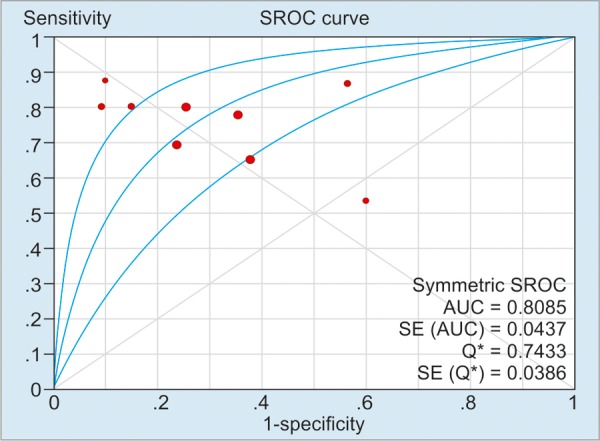
Summary receiver operating characteristic curve of FC assay in monitoring CD relapse at a cutoff value of >150 μg/g. Red spots in the figure denote the included studies of this pooled analysis

As illustrated in Table 2, the pooled sensitivity and specificity of included studies for CD activity were 91% (50 μg/g), 84% (100 μg/g), and 76% (>150 μg/g) respectively; and the pooled sensitivity and specificity of included studies’ values for CD relapse were 72% (100 μg/gm) and 75% (>150 μg/gm) respectively. For detection of heterogeneity, *I*^2^ was calculated, and the values of *I*^2^ for the sensitivity and the specificity ranged from 0 to 81.5%, indicating a relatively high risk of heterogeneity between the patients of included studies. The summary AUC of FC for detecting CD activity was 0.78 (50 μg/g), 0.88 (100 μg/g), 0.85 (>150 μg/g); for monitoring relapse of CD was 0.69 (100 μg/g) and 0.81 (>150 μg/g) respectively. Graphs 1 to 5 show the hierarchical SROC graph with the 95% confidence region and the 95% prediction region. With regard to CD activity, the summarized positive likelihood ratio (95% CI) of FC assay at cutoff values of 50, 100, and >150 μg/g were 1.68 (1.35–2.07), 2.38 (1.34–4.25), and 3.51 (2.56–4.80) respectively; the summarized negative likelihood ratio (95% CI) of FC assay at cutoff values of 50, 100, and >150 μg/g were 0.188 (0.12–0.31), 0.24 (0.18–0.32), and 0.31 (0.17–0.57) respectively. The data for monitoring CD relapse are presented in Table 2.

**Table 2:** **Table 2:** Different cutoff values of FC for predicting or monitoring activity or relapse of CD

		*CD activity*						*CD relapse*			
*Items*		*50 μg/g*		*100 μg/g*		*>150 μg/g*		*100 μg/g*		*>150 μg/g*	
Sensitivity (95% CI)		0.91 (0.87–0.95)		0.84 (0.80–0.88)		0.76 (0.69–0.82)		0.72 (0.57–0.84)		0.75 (0.67–0.82)	
*I*^2^ value (%)		71.3		10.5		80.4		0		4.0	
Specificity (95% CI)		0.47 (0.35–0.59)		0.61 (0.52–0.69)		0.78 (0.71–0.85)		0.56 (0.45–0.67)		0.71 (0.66–0.76)	
*I*^2^ value		19.6		77.7		0		70.9		75.5	
PLR (95% CI)		1.68 (1.35–2.07)		2.38 (1.34–4.25)		3.51 (2.56–4.80)		1.48 (1.05–2.09)		2.42 (1.65–3.56)	
*I*^2^ value		0		81.5		0		29.1		70.5	
NLR (95% CI)		0.188 (0.12–0.31)		0.24 (0.18–0.32)		0.31 (0.17–0.57)		0.58 (0.34–0.98)		0.38 (0.25–0.57)	
*I*^2^ value		0		8.1		69.6		0		39.5	
DOR (95% CI)		10.21 (5.08–20.51)		10.20 (5.36–19.41)		11.68 (4.61–29.59)		2.73 (1.19–6.26)		7.12 (3.24–15.65)	
*I*^2^ value		0		35.2		52.0		0		57.2	

FC: Fecal calprotectin; CD: Crohn’s disease; PLR: Positive likelihood ratio; NLR: Negative likelihood ratio; CI: Confidence interval

Next, we performed a subgroup analysis to assess the potential sources of heterogeneity between eligible studies. As calculated, the diagnostic accuracy of FC test was higher in European patients (DOR, 13.98) than in Asian subjects (DOR, 11.68). Similar results were observed in populations with the following characteristics: Larger sample sizes (number of enrolled patients over 30), the different kit for FC assay (other ELISA kit *vs* PhiCal kit). In addition, a meta-regression analysis was carried out to find whether there were any significant sources of heterogeneity. The findings revealed that testing FC using different detection kit was significantly associated with the accuracy of endoscopies for the activity of CD (p = 0.03).

To assess the effect of individual study on the summarized accuracy of FC test for detection of CD activity at a cutoff value of 50 μg/g, we also performed a sensitivity analysis. The summarized sensitivity, specificity, DOR, and AUC with 95% CIs were calculated by removing each individual study (Table 3). The results demonstrated that the diagnostic accuracy of FC for detection of CD activity was relatively stable.

**Table 3:** **Table 3:** Sensitivity analysis of FC prognostic value by excluding each included studies

		*Excluded from CD activity*						*Excluded from CD relapse*					
*Items*		*Langhorst et al^21^*		*Sipponen et al^10,22^*		*Schoepfer et al^5^*		*Guidi et al^33^*		*Lasson et al^14^*		*Laharie et al^32^*	
Sensitivity (95% CI)		0.90 (0.84–0.94)		0.91 (0.85–0.95)		0.94 (0.88–0.98)		0.72 (0.55–0.86)		0.67 (0.48–0.82)		0.78 (0.56–0.93)	
*I*^2^ value (% )		0		85.6		79.2		39.7		0		0	
Specificity (95% CI)		0.50 (0.37–0.63)		0.50 (0.33–0.67)		0.41 (0.27–0.57)		0.43 (0.27–0.59)		0.62 (0.49–0.74)		0.60 (0.46–0.72)	
*I*^2^ value		5.9		55.8		0		0		66.9		83.2	
PLR (95% CI)		1.77 (1.38–2.28)		1.72 (1.16–2.55)		1.57 (1.23–2.00)		1.28 (0.93–1.77)		1.66 (0.90–3.05)		1.67 (0.94–2.95)	
*I*^2^ value		0		40.6		0		0		58.0		57.7	
NLR (95% CI)		0.20 (0.12–0.32)		0.18 (0.10–0.35)		0.17 (0.08–0.39)		0.66 (0.35–1.24)		0.61 (0.34–1.07)		0.43 (0.19–0.96)	
*I*^2^ value		0		1.4		0		0		0		0	
DOR (95% CI)		9.61 (4.69–19.66)		11.69 (4.64–29.44)		9.81 (3.59–26.81)		2.03 (0.76–5.48)		2.77 (0.91–8.43)		4.26 (1.34–13.57)	
*I*^2^ value		0		0		0		0		27.7		0	

PLR: Positive likelihood ratio; NLR: Negative likelihood ratio

## DISCUSSION

In this article, we have studied the diagnostic accuracy of FC for detection of activity and relapse of CD. To our knowledge, this is the first meta-analysis focusing on the performance of FC in predicting both activity and relapse of CD. The systematic search of relevant literature identified 18 observational studies assessing the diagnostic performance of FC for detection of CD activity and recurrence that met the inclusion criteria and provided sufficient data to conduct a meta-analysis. The findings of our meta-analysis proved that FC at different cutoff values is not highly accurate for CD activity, pooled sensitivity is around 0.75, and summary specificity is between 0.47 and 0.78. For relapse of CD, pooled sensitivity is around 0.74, and summary specificity is between 0.56 and 0.71. The estimated accuracy of FC for assessing activity of CD might be overestimated according to the significant indication of pooled asymmetry. Furthermore, as the quality of included studies was not consistent, it is not easy to figure out whether the insufficient diagnostic accuracy reflects a problem with the quality of the information used in this analysis.

Endoscopy and imaging examinations are the preferred methods for evaluating CD activity. However, there are several disadvantages and risks when applying these methods. Endoscopy is invasive and needs bowel cleansing. Imaging techniques, such as magnetic resonance imaging are expensive and require intravenous contrast in most cases. Nowadays, numerous biomarkers are recommended for monitoring intestinal inflammation and therefore could also be used as potential indicators of CD activity. However, the clinical evidence supporting their application in the prediction and management of CD is still insufficient. Among the various factors, CRP has been proved to be a good responder in CD, but its application is limited due to its modest accuracy in predicting CD activity.^27^ As CD is a disease of digestive tract, fecal biomarkers have been supposed to be more accurate in assessing disease activity, and FC is mostly studied. Fecal calprotectin accounts for 60% of cytosolic proteins in granulocytes, and its concentration in feces is therefore proportional to neutrophil migration to the gut and reflects the potential degree of inflammation.^28^ In addition, FC is considered to be a promising biomarker for CD as it is stable in the feces, and its measurement is simple and noninvasive. To date, there is no specific meta-analysis emphasizing on the predictive performance of FC on both activity and relapse of CD. But a few meta-analyses^34,35^ have shown that FC is useful for prediction of activity or relapse of patients with IBD. The study of Lin et al^35^ evaluated the diagnostic accuracy of FC for discriminating patients with active IBD and those in remission according to 13 studies. In their analysis, the AUC value of FC was 0.89 for patients with IBD, 0.88 for patients with CD. The summary specificity for CD was 0.81. For the IBD group at a cutoff value of 50 μg/g, the pooled sensitivity was 0.92 and specificity was 0.60. For a cutoff value at 100 μg/g, the pooled sensitivity was 0.84 and specificity was 0.66. For a cutoff value at 250 mg/g, the pooled sensitivity was 0.80 and specificity was 0.82. However, they did not report the pooled data of sensitivity and specificity at different cutoff values for CD. Another meta-analysis also studied the potential role of FC in IBD patients. In another study, Mao et al^34^ performed a meta-analysis to evaluate the predictive capacity of FC in IBD relapse, but they failed to fully explore the predictive value of FC in CD patients as insufficiency of available data. They reported a value of 0.75 for sensitivity and 0.71 for specificity in patients with CD. In the present meta-analysis, we only included CD patients. The diagnostic accuracy in our study is similar with that reported by other investigators.

In this meta-analysis, the results indicated that the diagnostic value of the FC test is high for CD patients. However, as mentioned by other researchers, we cannot just simply use the FC assay to evaluate CD activity or treatment efficacy. Fecal calprotectin should be used in combination with other tests, such as ESR and CRP to assess the potential inflammatory condition of patients with CD. High FC levels with low CDAI scores may require other examinations to evaluate the condition of CD. And the measurements of FC should be performed in a periodic interval.

Though we tried to avoid any possible bias, there are still several limitations in our meta-analysis. First, the pooled results of the present study had relatively high heterogeneity and bias of publication. The sources of heterogeneity and publication bias include differences in populations, disease duration, treatment regimen, time of measuring FC, and cutoff value of FC. Second, the samples of included studies varied significantly. The pooled results were probably compromised due to the small number of eligible studies. Third, the reference standard was similar in most included studies, but the application of various scoring systems to assess CD activity was a problem. Therefore, a standardized scoring method should be established to minimize variation and provide a better accuracy. Last, a few studies failed to provide sufficient data or information of interest.

## CONCLUSION

In summary, this meta-analysis showed that FC is capable of monitoring activity and relapse of CD. The use of FC in assessing CD activity and relapse may provide a convenient, noninvasive way to enhance the management of CD before, during, and after treatment and, therefore, benefit patients with CD from the clinical outcomes. However, more elaborate and precise investigation of FC for predicting CD activity or relapse is needed.
